# Supplementary material for: Estimating axial diffusivity in the NODDI model
Source: Neuroimage. Author manuscript; Available in PMC 2022 Dec 30. (PMC9802007; doi:10.1016/j.neuroimage.2022.119535)
Supplement: Supplementary material [file NIHMS1860650-supplement-Supplementary_material.pdf]

Supplementary material

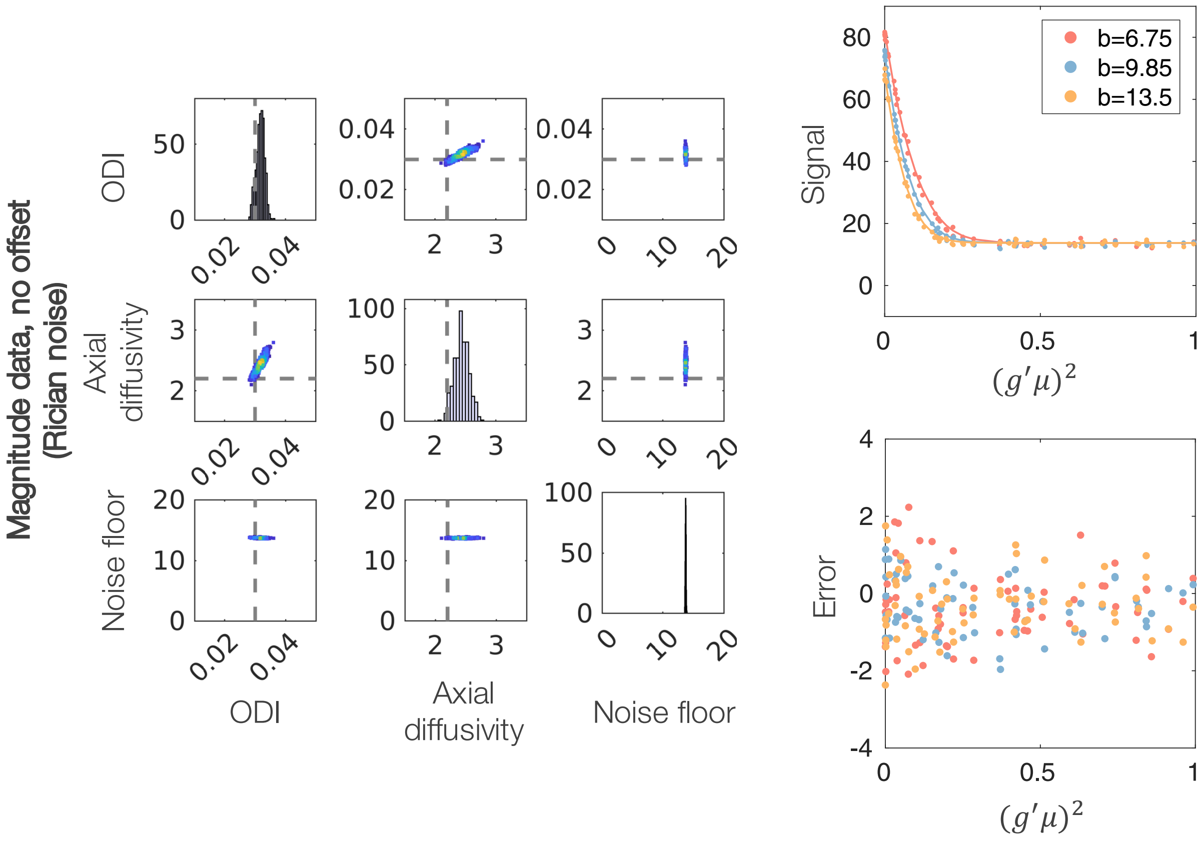

Supplementary Figure 1: Left: Parameter distributions output from MCMC for magnitude data in a simplified model where the signal offset $c = 0$ and c is not estimated as a parameter of the model. Each data point in the scatter plots represents a combination of parameters that fits the signal equally well. Grey dashed lines represent ground truth values. Right: Model fit (line) to simulated data (dots) and the associated error (prediction-data). g is the gradient direction, μ the fibre orientation and b the b-value in ms/μm^2. The signal is averaged over 100 voxels, each with SNR=16.5.
